# Disease of the Antrum

**Published:** 1871-04

**Authors:** Edward W. Anderson


### ARTICLE III.

### *Disease of the Antrum.*

By Edward W. Anderson, D.D.S.

In July last I was consulted by a gentleman I had known from boyhood, and who belonged to a family in which phthisis pulmonalis was hereditary. One by one had I watched his brothers and sisters, his father and mother, numbering originally perhaps six or eight, fall victims to this terrible disease, until all but he were dead. He then was pale and sickly looking—of about thirty years of age, and of a decidedly scrofulous cast. His cheek was so sunken and his form so emaciated, that one would have supposed at first sight that some frightful disease was preying upon his vitals, and required but a few more weeks to complete its work and lay him in the grave with his kindred. He was not long in making known his sufferings, and I, by asking various questions, learned from him the following facts:

He, while in Baltimore some two weeks before, had a tooth extracted, (the right superior second molar.)

The removal of this was followed by a dark, bloody discharge which was disagreeable to the taste, and continued at times for two days, when it ceased. Soon afterwards the right side of his nose became sore; the inner corner of his eye inflamed; the cheek more or less sensitive to the touch and a slight pain on the side of the neck below the ear. In a day or two a profuse discharge of fetid pus began to pour from the right nostril and with this the above symptoms began to subside.

He went to a physician for advice and was informed that he was troubled by catarrh, which, with some prudence on his part, would get well of itself. He, however, thought he had better take muriate tinct. of iron three times a day. He consoled himself with this belief for a time, but finding that he was growing worse, and that the discharge was becoming more profuse and fetid, he determined to consult another physician. He did so, and learned from this gentleman that his disease was ozena, and that an injection up the nostril was necessary.

He, from some cause or other, loosing faith in this decision, thought "he would visit me 'inasmuch as I was just from College, and hear what I would have to say about it, even if I had not put up my 'shingle.'"

I declared his disease to be inflammation of the lining membrane of the antrum, and advised him to consult Professor Gorgas, of the Baltimore College of Dental Surgery. He determined to do so, but on his arrival in the city he learned that Prof. Gorgas was absent from home.

This compelled him to seek aid elsewhere. He went to the dentist who extracted the tooth. This gentleman found on probing the palatine root that it penetrated the sinus, and he injected it copiously through this opening, with tepid water. More than a tablespoonful of purulent matter was discharged through the nose, the removal of which gave much relief to the patient. The dentist gave him a wash (unknown to me) to be injected three times a week, and a physician gave him some pills to improve his general health. With these he returned home and requested me to do the injecting for him, a request I readily granted, because I was anxious to witness the different stages of the disease. As soon as the antrum was emptied, the constant dripping from the nose ceased, but every injection proved an accumulation of pus in the cavity, a fact which lead me to advise a more frequent use of the syringe. He readily agreed, and for more than a week every thing "moved on as smoothly as a marriage bell," and he began to believe himself almost well, in spite of my opinion to the contrary, and constant warnings against cold and exposure. He accordingly resumed his old habits of riding late at night, and, if occasion required, in the rain; and as a reward for this imprudence, contracted a severe cold. This irritated the antrum beyond measure, and its secretions were increased surprisingly. In order to prevent the pus from dripping from the nose, I had to inject morning and night during four or five days; but as the cold yielded to remedies the antrum got better and hope again revived.

From this my patient began to be extremely imprudent. In damp and chill as well as in sunshine, he could be found attending to his duties, and no influence of mine could keep him within bounds, and in consequence his disease gradually grew worse, and at the expiration of the sixth week he became very dispondent.

Notwithstanding he had not used the care he should have exercised in order that the remedies he had been using might act without hinderance, I thought I could see that they were not equal to the emergency, and determined to change the treatment. I gave him Professor Gorgas' favorite prescription :

℞—Iodide of Iron, gtt. x.
Iodide of Potassa, gr. viii.
Cod Liver Oil, one dessert spoonful.

Take 3 times a day, ½ hour before meals.

Inject antrum with Permanganate of Potash.

This prescription acted like a charm. Notwithstanding he kept up his old habits of exposure on his farm, he began to improve gradually. Cold after cold was taken, but the effects of each succeeding one were less and less apparent. The orifice in the mouth began to assume a healthy appearance and to close, and his general health was much improved. In November, when, on the eve of starting to Baltimore to assume my studies at the Dental College, when I bade him adieu, he told me that he had not felt so well for years. Eight weeks afterwards he wrote to me as follows:

"My antrum is perfectly well. I can never express my deep obligations to you for effecting its cure, but hope the day is not far distant, when I will have an opportunity to act what I cannot now find words to express."

---

